# A Ready Test for Arsenical Compounds

**Published:** 1877-04

**Authors:** Edward Gaillard


A Ready Test for Arsenical Compounds.—*By Edward Gaillard, Ph.G.*—Who is the pharmacist that has not been called upon by his patrons or the physicians to know, at once, if this powder or that liquid did not contain ratsbane or arsenic, and often been obliged to make some excuse for the lack of knowledge, or felt the want of a more simple and ready test for the detection of arsenic, than the old, time-honored one of Marsh's. If we have the apparatus, or extemporize one, are we positive that it is free, at all times, of traces of that metal from previous operations? Besides it labors under many serious disadvantages: first, that sulphuric acid; secondly, that metallic zinc, which are employed in the test, may, one or other of them, or even both, contain more or less of arsenic as an impurity, and thus the indications of that substance obtained may not be due to its existing in the suspected matter underinvestigation. I may add that it is difficult to get, in commerce, zinc and sulphuric acid perfectly free from arsenic.

The test proposed by Edmund W. Davy, professor of forensic medicine in the Royal College of Surgeons, of Ireland, is one of such simplicity, and has proved so practical in my hands that I would recommend it to the pharmacist desiring to be *Probus Paratus*, always ready, at the instant, to decide at once when life and death depend upon his knowledge, and is so easy of execution that it may be performed by almost any one, and found practical for the object stated, especially to those who are not conversant with the details of chemical manipulation. It is a modification of Marsh's test, a well known method, and is founded on the circumstances that nascent hydrogen, in the presence of certain compounds of arsenic, will give rise to the formation of arsinuretted hydrogen; and thus very minute quantities of arsenic, under different circumstances, can be readily detected.

The modification used is the employment of an amalgam of sodium and mercury as a means of generating the hydrogen required for the test, and by the use of this substance do away with, altogether, the necessity of any acid, and employ two metals which are not liable to arsenical contamination. As to sodium, arsenic has never been pointed out as one of its impurities, and as to its presence in mercury, that is a circumstance of very rare occurrence; should it exist in that metal as an impurity, it can be readily removed from it by digesting the mercury in dilute nitric acid, and afterwards well washing it with water.

The amalgam found to answer best for the test consists of one part, by weight, of sodium to eight or ten parts of mercury, and is easily made by heating, moderately, in a test-tube, over a lamp, the mercury, and then adding gradually, in small pieces, the sodum, taking care to keep the mouth of the tube away from the face, if unprotected, lest some of the metal, in an ignited state, might be spurted out during the additions of the first portions.

The metals combine readily under these circumstances, forming an alloy that is liquid whilst hot, but becomes hard and brittle when cold. The contents of the tube, while still hot and liquid, are quickly poured out on a clean plate, when cool broken up for future use, and immediately placed in a stopped bottle. The way to employ this amalgam is simply to place the suspected matter, or solution, along with a little water, in the bottom of a test glass or a tumbler; then add a small bit of amalgam, about the size of a grain of wheat; and, lastly, place, without delay, on the top of the glass a piece of white filtering paper or the cover of a white porcelain crucible, moistened with a drop of a dilute solution of nitrate of silver, slightly acidulated with nitric acid, when, if arsenic is present, a dull black or deep brown stain on the paper, or a dark silvery one on the porcelain, will be quickly developed in the part moistened, owing to the silver of the salt being reduced to a metallic condition by the arseniuretted hydrogen thus evolved.

The silver solution, found to answer well for this purpose, is made by dissolving 20 grains of the nitrate in an ounce of distilled water, adding 2 drops of nitric acid, to render the solution slightly acid. Exceedingly minute quantities of arsenic can be readily detected by this very simple process. Thus one one-thousandth part of a grain of arsenious acid dissolved in 1 cc. of distilled water gives a very decided effect in a few moments, and even a smaller quantity can be detected; as, for example, one drop of Fowler's solution in an ounce of water will indicate in a little time by the blackening of the silver salt. I may further state that the presence of organic matter seems to interfere but little with this test; for I have found that very minute quantities of arsenious acid, when mixed with considerable amounts of milk, tea, coffee, ale or porter, or flour, could, with almost the same facility, be detected by this method, showing the applications are very extended.

Antimony is the only metal which is capable of uniting with nascent hydrogen to form a gas (antimoniuretted hydrogen), which, coming in contact with nitrate of silver, produces black antimonide of that metal; and the blackening of the silver salt from the formation of that compound might be easily mistaken for the effect produced by the arsenical gas.

The fact, first pointed out by Fleitmann, that antimoniuretted hydrogen is not evolved (except, perhaps, as a mere trace) from strongly alkaline solutions, though the conditions may exist there for its formation, and as the action of the sodium amalgam is to render the mixture quickly alkaline, there will be only a very minute quantity of antimony that may be present so evolved; and by previously rendering the mixture strongly alkaline, we may altogether prevent the evolution of that gas.

If, however, we make the mixture containing the antimony in solution first strongly acid, and then add the amalgam, or even acidify after its addition, the antimoniuretted hydrogen will be evolved in abundance, producing a deep black stain on the paper moistened with the nitrate of silver, and, for the purpose of this acidification, tartaric acid answers very well.

As the presence of alkalies in solution does not interfere with the evolution of the arsenical gas, this itself is a means of distinguishing the two metals, arsenic and antimony.—*Am. Journal of Pharmacy.*

